# South African podiatry students’ perceptions of feedback given as part of clinical training

**DOI:** 10.1186/s13047-018-0279-9

**Published:** 2018-07-03

**Authors:** Simiso Ntuli, Noleen Nomthi September, Nozipho Sithole

**Affiliations:** Department of Podiatry, Faculty of Health Sciences, University of Johannesburg, P O Box 524, Auckland Park, Johannesburg, Gauteng 2006 South Africa

**Keywords:** Podiatry clinical training, Podiatry clinical supervision, Podiatry students, Feedback in podiatry training, Positive &negative feedback

## Abstract

**Background:**

As part of their clinical training podiatry students spend time in clinical settings treating patients under the supervision of qualified podiatrists. The role and purpose of feedback during such clinical training is to improve students’ knowledge, skills and behaviour. Feedback is an integral part of the learning process that should enhance students’ clinical learning experiences. However, there is no data on podiatry students’ satisfaction or lack thereof about feedback provided during clinical training. The aim of this study was to determine the perceptions of podiatry students on feedback given or received during clinical training.

**Methods:**

Cross-sectional survey design study in which a four-section self-constructed questionnaire was used to collect data from podiatry students in their 2nd to 4th -year of study. Simple descriptive statistics were used to analyse quantitative responses with free text comments yielding qualitative data, which has been used to give more insight into the quantitative findings.

**Results:**

Analyses showed that 8% of students were satisfied, 52% were sometimes satisfied and 37% were not satisfied with the feedback. The majority (86%) of students indicated they would prefer to receive feedback in private. Seventy-three percent of students received positive (reinforcing) and negative (corrective) feedback at the same time.

**Conclusion:**

Students agree that feedback is an essential component of the clinical learning process and appreciate constructive regular feedback whether negative or positive in nature. Additionally, students understand that feedback regardless of its type has the potential to identify areas of development, reinforce good practice and motivate them to work toward their learning outcome expectations. However, there is a need to consider issues such as setting and timing when giving feedback.

## Background

In a clinical medical education setting, feedback refers to information describing students’ performance in a given activity [1]. Feedback should be ongoing, designed to be immediate and to provide direction and guidance. Feedback is a key step in the acquisition of clinical skills, yet it is often omitted, transmitted or received in a less than satisfactory fashion during clinical training [1]. Podiatry students learn clinical skills such as taking a patient history, performing a physical examination, and diagnosing, which represents a collation of cognitive and psychomotor skills and behaviours. Students learn these skills more rapidly when observed or demonstrated, rather than when described. Thus, feedback plays a crucial role in student learning in clinical training by encouraging students to reflect on their performance and on ways of improving it in order to reduce discrepancies between actual and desired performance [2]. Clinical training is a crucial aspect of teaching and learning in any clinical domain, as it allows students to demonstrate their clinical competence. Therefore, clinical training enables students to use theoretical and conceptual knowledge to develop the requisite podiatric clinical skills required for independent clinical practice [1]. In a profession like podiatry, feedback on performance in during clinical training is extremely important for the development of competent independent practitioners.

Accordingly, it is international practice that podiatry students treat patients as part of their undergraduate training; at the University of Johannesburg (UJ) podiatry, students begin treating patients in their second year of study. During these clinical sessions, qualified podiatry clinicians provide supervision and mentoring of students, which is fundamental in supporting clinical learning. The majority of supervising clinicians are full time clinicians employed at the clinical training sites and supervise students on a part time basis. These clinicians have no teaching and/or assessment background, which may affect their capacity as clinical supervisors [3]. However, the training institution does provides an annual one-day supervisor induction and training workshop to all supervising clinicians.

Evidence has shown that formative feedback is essential [1] and instrumental in the development of students [4]. Skills should be enhanced through feedback that is constructive [5]. Feedback is an informed, non-evaluative and objective appraisal of performance aimed at improving clinical skills [6]. It is “an interactive process which aims to provide students with insight into their performance” [1]. It is vital for the progression of the student and should be an integral part of teaching [7]. Students welcome feedback and maintain more interest when it is provided in a constructive and implementable manner [8]. High-quality feedback is associated with students’ maintenance of interest and perceptions of high-quality teaching [9–12].

It is important to keep in mind the link between effective feedback and students’ motivation and self-esteem, as improvement in clinical practice comes from the student’s motivation to do well and is supported by the knowledge gained from the feedback given [13]. For feedback to be effective, literature suggests that it must focus on the student, concentrate on important points, [4, 14] be constructive, [5] situational [15] timely, specific and most importantly non-judgemental [6, 14]. Clearly then, feedback has to be carefully constructed by the clinicians, well planned and of high quality if it is to have a positive effect on students’ learning.

In addition, prior to giving feedback, barriers to effective feedback must be identified and addressed accordingly. These barriers may include the complexity of clinical cases [16], time [17, 18], emotional aspects such as student’s confidence, motivation and self-esteem as well as the clinician-student relationship [13].

Clinicians should keep in mind the primary goal of clinical training, which is to provide students with opportunities to practice and become proficient in the knowledge and skills essential for professional podiatry practice. Thus, both students and clinicians must understand that feedback is the cornerstone of effective clinical teaching and that receiving accurate and timely feedback can help narrow the gap between actual and desired clinical performance [19]. Moreover, for good practice to be reinforced and poor performance corrected, effective feedback is necessary.

As part of their supervision, clinicians are expected to assess and then provide feedback to students. Despite an extensive search of the literature, no empirical data could be found about the perception of podiatry students on feedback received during clinical training. The aim of this study was to investigate podiatry students’ perception of feedback given during clinical training.

## Methods

### Study design and participants

A cross-sectional survey design study was used in this mainly quantitative methods study. A free text section was included after each question to provide qualitative responses should the participant wish to do so.

The target population for the study was all registered podiatry students in their 2nd, 3rd or 4th years of study at the UJ. At the time of the study, there were 101 registered students in the selected years of study.

Only students over the age of 18 years and in their second, third or fourth years of study were eligible to participate. First-year students were excluded, as they do not yet have clinical placement as part of their training.

### Ethical approval

The Research Ethics Committee of the Faculty of Health Sciences, University of Johannesburg (REC-241112-035), gave ethical clearance for the study.

Students are regarded as a vulnerable group. Thus, prior to using them in a research study, permission was obtained from the Director, Division for Institutional Planning, Evaluation and Monitoring (DIPEM). After obtaining permission from DIPEM, students in the selected years of study were approached to request them to participate in the study. Each participant gave consent and signed an informed consent form before any data collection took place. To ensure students’ confidentiality and anonymity no demographical data was collected except for each student year of study.

### Data collection procedure

Data collection was undertaken using a four-question self-constructed questionnaire. The questionnaire was designed based on anecdotal experiences of the authors (two academics and one final year student). During the discussion on the design of the questionnaire, one author (student) raised the issue of anxiety as a factor that affects clinical performance. The clinical training environment for undergraduate students can present challenges that may cause students to experience stress and anxiety [20]. High levels of anxiety can affect the students’ clinical performance, presenting a clear threat to success in clinical training; therefore, in this study anxiety was included as one of the factors that may affect feedback.

As the questionnaire was self-constructed before being used in the study, it was piloted with five students (two from 2nd and 3rd year and one from 4th year) selected by convenience sampling across the three years of study. The students on whom the questionnaire was piloted were subsequently excluded from the study. Following this process, no changes were required and the questionnaire was utilised for data collection. The questionnaire covered the following domains; *satisfaction with feedback*, *type of feedback received, possible reasons for the type of feedback received* and *preferred setting to receive feedback*. Students were given a choice to provide qualitative data in the form of free text after each question, in order to give a detailed understanding of their responses.

Data collection was over a two-week period in August 2016. The researchers distributed one copy of the questionnaire to each student after a signed informed consent form was received in the selected years of study during lecture time. The respective year coordinators were requested to give students 15 min to compete the questionnaire before starting with the lecture. To ensure privacy, students dropped their completed questionnaires in a lockable box placed at the Podiatry clinic reception area.

### Data analysis

Descriptive statistics were used to characterize the study sample and to provide simple summaries of the quantitative responses.

Inductive coding was utilised to identify free codes, which were subsequently collapsed into themes where a relationship existed. Thematic analysis, which is a search for themes that emerge as being important to the description of the phenomenon, was used to analyse free text data in this study. This involved the identification of themes through “careful reading and re-reading of the comments” to capture an aspect of the students’ perception of feedback.

## Results

Eighty-five (85) questionnaires were received from the 96 distributed indicating a response rate of 88.5%. The majority of students who participated in the study were in their 2nd year of study.

**Fig. 1:**
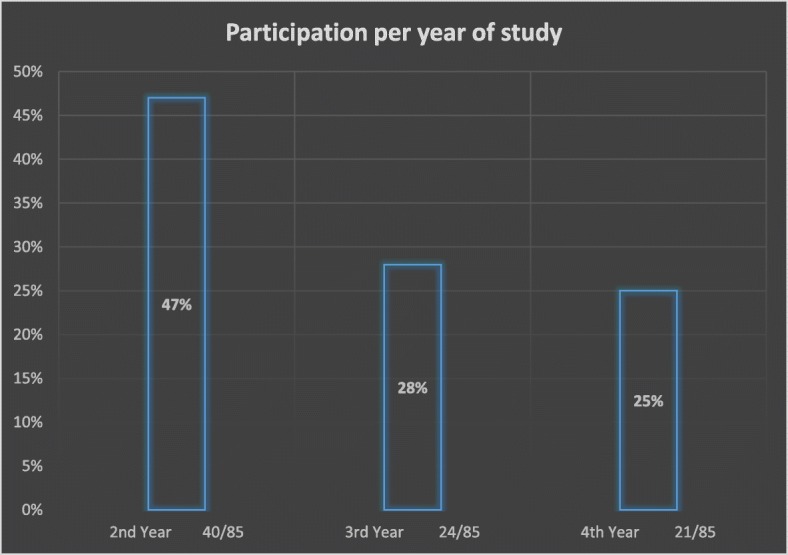
Participation. Percentage of participants per year of study, students in their first year of study were excluded in this study

Figure 1 provides the details of participation per year of study.

Table 1 Overall 8.2% *(7/85)* of the students indicated that they were completely satisfied with feedback, 51.8% *(44/85)* were satisfied sometimes and 37.6% *(32/85)* were not satisfied the remaining 2.4% *(2/85)* were not sure.

**Table 1:** Satisfaction with feedback

			Yes	No	Sometimes	Not sure	Total
Group	2nd year	Count	4	15	20	1	40
		% within Group	10,0%	37,5%	50,0%	2,5%	100,0%
	3rd year	Count	3	4	16	1	24
		% within Group	12,5%	16,7%	66,7%	4,2%	100,0%
	4th year	Count	0	13	8	0	21
		% within Group	0,0%	61,9%	38,1%	0,0%	100,0%
Total		Count	7	32	44	2	85
		% within Group	8,2%	37,6%	51,8%	2,4%	100,0%

Table 1 presents the findings per year group.

Table 2 Six percent *(5/84)* of students indicated they received positive feedback, 17.9% *(15/84)* received negative feedback and 76.2% *(64/84)* of students received positive and negative feedback simultaneously.

**Table 2:** Type of feedback frequently received

			Negative	Positive	A bit of both	Total
Group	2nd year	Count	6	3	31	40
		% within Group	15,0%	7,5%	77,5%	100,0%
	3rd year	Count	3	2	18	23
		% within Group	13,0%	8,7%	78,3%	100,0%
	4th year	Count	6	0	15	21
		% within Group	28,6%	0,0%	71,4%	100,0%
Total		Count	15	5	64	84
		% within Group	17,9%	6,0%	76,2%	100,0%

Table 2 presents the findings per year group.

Table 3 The majority of students 55.3% *(47/85)* cited anxiety as a factor influencing feedback, 12.9% *10/85)* cited their lack of clinic preparation, 12.9% *(11/85)* of the students mentioned being personally well prepared was a factor, and extra reading was noted as being beneficial in the remaining 20% *(17/85)*.

**Table 3:** Factors perceived as influencing the type of feedback received

			Lack of preparedness for clinics.	Anxiety.	Being well prepared.	Extra reading.	Total
Group	2nd year	Count	5	24	6	5	40
		% within Group	12,5%	60,0%	15,0%	12,5%	100,0%
	3rd year	Count	5	13	3	3	24
		% within Group	20,8%	54,2%	12,5%	12,5%	100,0%
	4th year	Count	0	10	2	9	21
		% within Group	0,0%	47,6%	9,5%	42,9%	100,0%
Total		Count	10	47	11	17	85
		% within Group	11,8%	55,3%	12,9%	20,0%	100,0%

Table 3 presents factors that students thought influenced feedback received.

Table 4 The majority 85.9% *(n = 73/85)* of students’ preferred receiving feedback in private, 8.2% *(n = 7/85)* were okay with receiving feedback in front of classmates and 3.5% *(n = 3/85)* were okay with receiving feedback in front of a patient.

**Table 4:** Preferred setting to receive feedback

			In front of class mates	In private	In front of the patient	Other	Total
Group	2nd year	Count	2	35	2	1	40
		% within Group	5,0%	87,5%	5,0%	2,5%	100,0%
	3rd year	Count	4	19	0	1	24
		% within Group	16,7%	79,2%	0,0%	4,2%	100,0%
	4th year	Count	1	19	1	0	21
		% within Group	4,8%	90,5%	4,8%	0,0%	100,0%
Total		Count	7	73	3	2	85
		% within Group	8,2%	85,9%	3,5%	2,4%	100,0%

Table 4 presents the findings per year of study.

Qualitative findings

**Table:** 

Theme	Statement/s
Manner in which feedback is given.	*“I have felt more moaned at than constructively criticised… and it makes us anxious …, as feedback can be harsh and unenjoyable”.* *“Negative feedback related to my clinical performance assists me. However, clinicians need to learn to keep it clinical, specific and not personal which makes me feel angry and frustrated”.* “Some clinicians give feedback in a very disrespectful manner they shout* 1 *and this is discouraging and humiliating in front of a patient”.*
Confusion when negative (corrective) and positive (reinforcing) feedback is given concurrently.	*“Confusing because when one receives positive feedback you feel like you are doing something right, however, negative feedback has the opposite effect especially when given at the same time”.* *“Positive feedback encourages me to continue with the good work and negative feedback makes me want to learn more if given to me in a good way”.* *“I am able to see my mistakes, especially with negative feedback. Positive feedback is great and encouraging”.*
Need for privacy when receiving feedback.	*“A private setting will allow for a one on one, for me to ask questions on how to improve; makes me feel like I’m being addressed as an adult; ensures my more positive response the next time even if the feedback was negative”.* *“I prefer receiving feedback in privately so I can raise my issues and concerns without the fear of being laughed at by my classmates and to avoid losing trust from my patient”.* *“To have enough time to discuss feedback and not be rushed and have feedback that is individually focused”.*
Students’ appreciation of feedback.	*“When delivered a correct way, negative feedback motivates me to read up on whatever it is that I couldn’t master”.* *“It makes me determined to work harder than I did in order to receive positive feedback in the future”.* *“I am able to see my mistakes, especially with negative feedback. Positive feedback is great and encouraging”.*

## Discussion

In this study, students were conscious of the value and role of both positive (reinforcing) and negative (corrective) feedback in their clinical training and were appreciative of receiving feedback. Students value feedback that enables them to move forward in their professional development. This is noted in the comment/s such as; *“I am able to see my mistakes, especially with negative feedback. Positive feedback is great and encouraging”.*

In this study, 8.4% of the students were completely satisfied, 53% were sometimes satisfied and 38.6% were not satisfied with feedback. The authors noted that 62% of final year students were not satisfied with feedback. This was interesting as it was noted that this group did the most extra reading (42.9%) and 71.4% indicated that they received negative (corrective) and positive (reinforcing) feedback concurrently. Final year students have a high and varied patient caseload. In this year of study, students are exposed to and subsequently observed across all domains of podiatry clinical practise. During their training students are required to perform specialised procedures including nail and minor skin surgery, wound care, management of paediatric, sports and geriatric patients. The authors postulate that the gap between theory and practice, being unprepared for practice and high expectations from both patients and supervising clinicians may be the cause of poor satisfaction with feedback. In this group, 47.6% identified anxiety as a factor influencing the type of feedback they receive. The clinical learning environment for undergraduate students has been identified as a source of significant stress and anxiety for students [21, 22]. A number of reasons may lead to anxiety during clinical training amongst the final year students including but not limited to, the gap between theory and practice, feeling unprepared for independent practice, the fear of making a mistake, and heavy workloads associated with course requirements [22].

During clinical training, students learn patient history taking, examination skills, as well as diagnostic and communication of information skills. Podiatric clinical teaching takes place in the course of routine podiatric clinical care where discussion and decision-making take place in real time, with teaching often centred on the analysis of actual patient care that the student had undertaken. During such training, clinicians focus on each student learning needs and competence in clinical practice. Therefore, giving feedback offers a valuable method for the clinicians to deepen students’ learning experience. Consequently, receiving accurate feedback can help students narrow the gap between actual and desired clinical performance [19]. Feedback is, therefore, a core component of clinical teaching and learning and promotes learning by informing students of their progress, observing learning needs and motivating students to engage in appropriate learning activities [23, 24].

However, only 8% of students were completely satisfied with feedback given during clinical training. Free text analysis provided the needed insight to this low percentage. Students identified the manner in which clinicians give feedback as the crucial area of dissatisfaction.

*“Negative feedback related to my clinical performance assists me. However, clinicians need to learn to keep it clinical, specific and not personal which makes me feel angry and frustrated”.*

Clearly, the manner in which feedback is given is important. Positive feedback is ordinarily pleasing, easy to give or receive, whereas negative feedback can be both difficult to give or receive and often disappointing. Therefore, when giving any type of feedback, clinicians need to be careful not to appear as judgemental, which may be difficult to avoid especially when giving negative (corrective) feedback. When giving negative feedback, clinicians should bear in mind that there is no way of informing a student of his/her clinical errors without provoking some degree of disappointment [25]. This requires the clinicians to be both sensitive and skilled at giving feedback [25].

During clinical training, clinicians may observe incorrect clinical practice and must bring it to the student’s attention and address it timeously. In such cases, negative feedback must be both accurate and provide constructive criticism for it to be valuable to the student. When negative feedback is given in this way, it is of high-quality and addresses specific areas students might perceive it as an accurate evaluation of their performance [26]. Clinicians clearly require skill and understanding of the feedback process, especially when the feedback is negative. This means that clinicians must give negative feedback that is constructive, specific, appropriate and critical yet non-judgemental [27].

In this study, 70% of students stated that clinicians gave negative and positive feedback concurrently. Students, however, find this approach confusing, demotivating, and subsequently feel frustrated.

*“Confusing because when one receives positive feedback you feel like you are doing something right, however, negative feedback has the opposite effect especially when given at the same time”.*

Student in the free text section of the questionnaire expressed the following:

*“It makes me determined to work harder than I did in order to receive positive feedback in the future”.*

Students are happy to receive negative feedback that is constructive, specific and clearly identifies areas of development. However, there is a need to educate students on how to receive and process feedback, as feedback will not always come as either negative (corrective) or positive (reinforcing) [28]. From the qualitative analyses, it would seem that students prefer to receive only negative or positive feedback during feedback sessions. This may be ideal, but unlikely in practice as in most cases, feedback may include elements of both negative and positive feedback. There could also be a lack of understanding because of the time lapse, as students probably would not remember all the incidents in a clinical session. It may be useful to first discuss the corrective feedback and then make it clear that the clinician is moving on to reinforcing feedback. Students must keep in mind that feedback usually targets a common goal, which is reinforcing (positive), or correcting (negative) performance [29]. Thus, students must direct their focus on the actual content and not necessarily the type of feedback. Acknowledging and understanding this fact may influence students’ perception of positive and negative feedback when given concurrently.

In addition, the differences between negative and positive feedback given should be evident to students. Positive feedback should focus on acknowledging and reinforcing exemplary clinical performance and must include specific examples of what the student has done well. When positive feedback is given in this way it is perceived by the students as supporting clinical learning and motivating them to repeat excellent performance and prompts them to seek more feedback [30, 31]. On the other hand, negative feedback must highlight areas of incorrect clinical practice and areas needing improvements. Specific examples of incorrect clinical practice and suggestions for correction/improvement must accompany negative feedback. When delivering negative feedback, clinicians must use a respectful, reassuring tone and precise, descriptive and unbiased wording [32, 33]. In other studies, students reported that negative feedback was constructive when it focused on specific performance accompanied by reasons why the performance was incorrect or faulty [34] and when it dealt with behaviour that the student was able to control or modify [35]. Negative feedback given in this way can enhance the clinician–student relationship and can lead to improvements in the students’ perception of feedback [30].

If feedback is regarded as a critical part of and/or a tool in clinical teaching, it is reasonable to consider the setting in which it is given. Therefore, clinicians must create a positive learning environment in order for feedback to be maximally effective [2]. Such an environment should promote the concept that the clinical supervisor and student are working together to help the student achieve the clinical learning outcomes, with an expectation that the clinician will observe, assess performance and give feedback in an atmosphere of mutual trust and respect. Creating such a learning environment should also include clinicians’ appreciation of the role of feedback in clinical training and the difference between assessment and feedback. Clinicians must ensure that feedback presents information, rather than judgment [33] and is an ongoing part of the instructional process given regularly to enhance clinical learning and training [23]. Therefore, clinicians must use feedback as a developmental and an integral part of the learning process to ensure that their students remain focused on reaching clinical learning goals. Assessment, on the other hand, is summative, and should thus be reserved for the end of clinical training, to provide a judgment about how well or poorly a student has met predetermined clinical outcomes.

Currently, clinicians frequently give feedback during clinic times and at times in front of patients and other students. We found that 86% of students preferred receiving feedback in private.

*“A private setting will allow for a one on one, for me to ask questions on how to improve; makes me feel like I’m being addressed as an adult; ensures my more positive response the next time even if the feedback was negative”*.

A private setting is ideal as it allows for a two-way feedback discussion in which the student can play an important role in assessing his/her own performance [36]. In addition, giving feedback in private will enable each clinician to give timely, honest, specific feedback and offer ways to improve and reinforce clinical performance. Students, in turn, will be able to raise their learning concerns without fear of being ridiculed or losing confidence.

### Limitations

This study has contributed new knowledge in the area of podiatry training; however, the study has a number of limitations. Firstly, data were collected from one training institution. Secondly, the use of negative and positive feedback terms may have led to students identifying negative as bad and positive as good. On hindsight, the use of corrective feedback instead of negative and reinforcing feedback instead of positive may have yielded more insightful responses. Thirdly, in this study, only students’ perceptions of feedback were investigated and not those of supervising clinicians as well. This is a significant limitation as feedback by its nature is a dynamic process that involves the senders (the clinician) and the receivers (the students). Therefore, the findings of the study are biased towards students’ perceptions and describe the experiences of students of feedback during clinical training. Additionally, this study did not statistically analyse differences between the groups, which would have added deeper insight to the current study.

## Conclusion

Feedback is a core part of clinical learning and teaching; clinicians need to consider how and when they give feedback to students to ensure they are not missing the learning opportunities that clinical training provides. There is paucity of data on students’ perception of feedback given during clinical training in podiatry. The current study has highlighted the need to consider the manner in which clinicians give feedback and to ensure privacy when giving feedback. There is also a need to educate students on how to receive, process and reflect on feedback received during clinical training.

Students agree that feedback is an essential component of the clinical learning process and appreciate receiving constructive regular feedback whether negative (corrective) or positive (reinforcing) in nature. Students understand that feedback, regardless of its type, has the potential to identify areas of development, reinforce good practice and motivate them to work toward their learning outcome.

## Footnotes

1 This was an isolated finding and reported only by one final year student.
